# Understanding Parental Role in Children’s Participation in Decision Making during Hospitalisation: An Ethnographic Study in Malaysia

**DOI:** 10.21315/mjms2023.30.3.13

**Published:** 2023-06-27

**Authors:** Lee Siew Pien, Elaine Haycock-Stuart, Ashikin Atan, Nur Ainsyafinaz Shamsuddin

**Affiliations:** 1 Kulliyyah (Faculty) of Nursing, International Islamic University Malaysia, Pahang, Malaysia; 2 School of Health in Social Science, The University of Edinburgh, Edinburgh, United Kingdom; 3 Kuantan Medical Centre, Pahang, Malaysia

**Keywords:** children, participation, decision making, parental role, ethnography

## Abstract

**Introduction:**

Despite a growing recognition internationally that children have a right to participate in matters that affect their lives, they are not always involved in decision-making processes concerning their health care. There is a lack of information on how parents influence children’s participation in this decision making. This study explored the roles parents assume in processes regarding their children’s participation in communication exchanges and decision making in a paediatric oncology unit in Malaysia.

**Methods:**

This study adopted a focused ethnography design within a constructivist research paradigm. Participant observations and semi-structured interviews were conducted with 21 parents, 21 children and 19 nurses in a paediatric oncology unit in Malaysia. All observation fieldnotes and interview recordings were transcribed verbatim. A focused ethnographic data analysis technique was performed to analyse the data.

**Results:**

Three themes emerged regarding parents’ roles in the communication and decision-making processes involving their children: i) facilitators of communication; ii) communication brokers and iii) communication buffers.

**Conclusion:**

Parents controlled decision-making processes concerning their children, while children preferred and welcomed parents as consultants in the decision-making processes regarding their health care.

## Introduction

The United Nations Convention on the Rights of the Child (UNCRC) (1) has become the most widely endorsed human rights treaty in history, ratified by over 190 countries. Malaysia ratified the convention in 1995 with some reservations. Many initiatives to realise and uphold the rights of children have been advanced as part of the strategies to achieve these goals, such as the introduction of the Child Act in 2001 and the withdrawal of some of its initial reservations to the UNCRC. Taking these influential changes into account, it is possible that Malaysians’ views on childhood might change as they become more aware of the child as an active subject (2).

The participation of children in decision making relating to their care is a concept that emphasises the autonomy of children. This involves information sharing and communication between nurses, children and parents. Research suggests that children’s participation in health care and service provision may lead to benefits such as better provision of information, opportunities to express feelings, development of confidence and competence, feelings of being valued, increased locus of control and increased adherence (3, 4). In contrast, lack of participation can have adverse consequences, such as increased fears and anxieties, reduced self-esteem, depersonalisation and feeling unprepared for procedures (4–6).

Globally, parents have a legal right to influence and be involved in decision making surrounding their children’s health care. Health policy accepts that services should be child-centred, and that children should be encouraged to be active partners in information sharing and decision making about their health and care and, where possible, be able to exercise choices. This ensures that health care is customised as much as necessary to meet the needs and preferences of children and their families. In Malaysia, as in most countries, parents are their children’s representatives until they can fully represent themselves. Hospital policy emphasises parents’ participation in their children’s health care decision making, thus ensuring that services are provided in accordance with their children’s needs and enhancing parents’ control over their children’s health care services.

Despite participation being a fundamental right for children, they are seldom involved in consultations or decision making. Even when children express a desire for involvement, they are often not supported. Previous studies have reported that parents have a significant influence on whether children’s efforts to participate are facilitated and supported in hospital settings (3–10). However, parental involvement in decision making is often not sufficiently implemented, so the parental role during hospitalisation can be demanding. The existing literature reveals a discrepancy in the data on the role that parents assume surrounding children’s participation in communication exchanges and decision-making processes. More knowledge is required to inform health professionals about how to promote children’s participation. Thus, the aims of this study were to explore parents’ roles in the participation of their children in decision-making processes related to their health care.

## Methods

This focused ethnographic study was conducted over a 6-month period at one paediatric oncology ward of a tertiary hospital in Kuala Lumpur. Data collection consisted of participant observations and face-to-face semi-structured interviews, which were conducted with 21 parents, 21 children and 19 nurses. A sample size of 61 was selected as a result of data saturation when no new information was discovered after analysing the observation and interview transcripts. Purposive sampling was used to collect data from various groups of participants with specific characteristics to better understand the parental role in children’s decision making. Participants were purposively selected on the basis that they met the study’s inclusion criteria: i) children who were aged between 7 years old and 12 years old, were admitted to the selected ward at the time of data collection, were willing and able to take part and had parental consent; ii) parents of child participants who accompanied their children in the ward and iii) nurse participants who provided direct nursing care to the child participants. Children under seven and those in critical conditions were excluded from participation, as were their parents. Participants in this study were selected because they fulfilled particular characteristics that enabled a detailed exploration and understanding of parents’ roles surrounding children’s participation in decision making.

Fieldwork began upon obtaining ethical approval from the Malaysian Medical Research Ethics Committee (MREC). Prior to recruiting participants, meetings were held with departmental managers and members of staff to provide information about the study. At the end of the meetings, the nurses were given a flyer and the participant information sheet. They were asked to provide their name, years of working in the ward and contact number if they were interested in participating in the study. The nurses were given at least 24 h before they were approached about their participation in the study. A list of potential child and parent participants was gathered from the nurse in charge. The first researcher then personally met those who fulfilled the inclusion criteria to explain the nature of the study, making it clear that their participation was voluntary and that refusing to participate in or withdrawing from the study while it was in progress would not affect their care in any way. The parents were given the participant information sheet and details about the research and invited to participate in the study. Age-appropriate information was given to the children. The purpose and implementation steps of the research were explained to the participants. After obtaining signed informed consent from each participant, observations and interviews were conducted. After parents agreed, their children were also asked for oral consent before participating in the study (11).

Non-continuous participant observations were conducted and were spread out throughout the fieldwork (12). In her participant observer role, the first author was not part of the ward team and did not perform any hands-on nursing duties. Whenever appropriate, she interacted with the participants and participated in non-legally defined roles as might be performed by a care assistant, such as talking and playing with the children, helping with meals, accompanying the children to various parts of the hospital (such as the radiology department and clinics) and assisting with the nursing procedures (dressing, blood taking and vital sign checking). The daily observations included all nursing activities pertaining to children, such as doing nursing rounds, providing medication, checking vital signs, performing dressings and taking blood specimens, in addition to informal tasks, such as making small talk during procedures and client consultations. Each time a nurse participant attended to a child participant, she observed the nurse-parent-child interaction to gain a first-hand understanding of how parents played a role in their children’s decision-making involvement.

Interviews were conducted in the form of formal and informal conversations with the participants. The informal interviews were conducted spontaneously with parents, children and nurses, while the formal interviews were conducted in a quiet room on the ward. Conversations were recorded using an audio recording device with the participants’ consent. Each interview lasted from 30 min to 90 min, depending on how much time the participants had and how much information they shared on each topic. Using a topic guide that was developed from previous research (3, 6, 7), the participants were asked the following: i) their understanding about children’s participation in decision making; ii) their preferences and iii) how they would support their child.

The observation fieldnotes and interview data were transcribed verbatim and analysed using focused ethnographic data analysis techniques (13). The analysis involved four inter-related stages: i) coding of descriptive labels; ii) sorting for patterns; iii) generalising and iv) memo writing. The transcripts were read and re-read for familiarisation of the participants’ stories before they were examined for meaning so that similar statements could be coded. The codes that adhered closely to the participants’ own actions and accounts that had commonalities were sorted into patterns and grouped into key themes. For generalising, the linkages between the emic meanings of participants and the researcher’s etic interpretations were identified, and then theoretical understandings that took both perspectives into account were constructed. Memo writing was used throughout the analysis process. Ideas or insights about the data, which were forms of coding that made connections between pieces of information, were written in the memo.

To assess the rigour of the research, the four indicators of credibility, dependability, transferability and confirmability established by Lincoln and Guba (14) were applied. All of the fieldnotes and interviews were recorded, and the text analysis files were saved to ensure the data’s dependability and confirmability. This study used purposive sampling, a detailed description of the research setting and the participants’ provision of contextual information about the fieldwork sites to determine the transferability of the research. The researcher observed and interviewed each participant to obtain credible data regarding their experiences in the context of the children’s participation in the decision-making process. During the data analysis process, the author and her PhD supervisors examined the implications of the original data, determined which categories best fit the original data and provided operational definitions (peer debriefing) to ensure credibility. Following completion of the initial data analysis, six participants (two from each group) were asked to indicate whether the analysis results accurately reflected their experiences (member checks). These three participants indicated that the findings of this study mirrored their experiences.

## Results

### Demographic Characteristics

A total of 61 participants (21 parents, 21 children and 19 nurses) were included in this study. The demographics of the parents are presented in Table 1. The child participants were aged between 7 years old and 12 years old and had been diagnosed with leukaemia. The nurse participants were aged between 26 years old and 45 years old. They had a range of years of experience, with the majority having served for longer than 5 years; six participants had less than 5 years of experience (31.6%), nine participants had between 5 years and 10 years of experience (47.3%) and four had over 10 years of nursing experience (20.1%).

### Theme 1: Facilitators of Communication

The role of parents in children’s participation in decision making was drawn from Gibson and colleagues’ (15) model of communication. The data analysis yielded three themes representing these roles: facilitators of communication, communication brokers and communication buffers.

The parent’s role as a facilitator of communication is conceptualised in a situation in which the parent encourages communication between the nurse and the child. The first scenario involved Lina, who acted as a facilitator of communication:

A nurse attending to a 12-year-old boy and his mother says that the doctor has changed one of the child’s medications to liquid form due to the unavailability of the tablet in the pharmacy. The nurse asks the mother if her child can take syrup medication without turning to the child. The child is quiet, listening to the conversation between the nurse and his mother. The mother responds that she is not sure and instructs the nurse to ask the child herself. Without explaining why his medication has been changed, the nurse asks the child if he is able to take syrup medication. With a smile, the child answers that the liquid form is usually for small children, and he is now 12 years old, but he agrees to take it. (Fieldnote, 4)

The above scenario illustrates how a parent played her role as a facilitator of communication when she forged communication between the nurse and her child. The nurse relayed information to the mother rather than directly to the child. The child listened in the background and overheard that his medication needed to be changed. The parent did not speak on behalf of her child but urged the nurse to ask the child himself. Thus, the nurse openly sought the child’s agreement about the medication and allowed the child to voice his preference.

### Theme 2: Communication Brokers

The parent’s role as a communication broker is conceptualised in a situation where the parent conveys information from the nurse to the child:

A nurse approaches an 8-year-old Chinese girl and her mother, and informs them that she is going to withdraw the child’s blood specimen and will remove the IV line, indicating the IV line on the child’s left hand. When the nurse tries to hold the central venous line (CVL) on the child’s chest, the child cries and screams, ‘waw waw waw’, pushing the nurse’s hand away. Seeing what is happening, the mother, in a very firm voice, informs her child (in Mandarin) about what the nurse is going to do. Hearing them speaking in their native language, the nurse seeks the mother’s assistance to inform the child that the blood specimen will be withdrawn from the CVL and will not puncture her skin. She also explains that she will remove the line after she finishes the blood-taking procedure. The mother repeats the information to her child in Mandarin. The child wants to confirm that the nurse will not puncture her skin by asking her mother, ‘Are you sure? Just now she said she wanted to take my blood?’ The mother responds in an angry voice, saying, ‘Yes, she will draw your blood, but from here (gestures to the CVL line), not puncturing your skin’. The child stops crying and appears calmer. She allows the nurse to proceed with the procedure. (Fieldnote, 17)

The above scenario illustrates how the parent reiterated the information from the nurse to the child so that it could be better understood. Initially, the nurse did not specifically direct communication to the child, but conveyed the information to the parent until the difficulty was evident. The child’s refusal could be related to the language barrier or perhaps to the use of medical terms and lengthy sentences. Subsequently, when the child exhibited her refusal of the scheduled treatment, the nurse and parent jointly intervened to overcome this barrier by endeavouring to gain the cooperation of the child. The parent appeared to have employed the role of a communication broker, in which she explained and repeated the information to her child by translating it ad hoc. She conveyed information on behalf of the nurse, which helped the child integrate what the nurse had said. This allowed the child to address her own agenda and cooperate with the treatment.

### Theme 3: Communication Buffers

The parent’s role as a communication buffer is conceptualised in a situation in which the parent falsifies information from the nurse for the child:

A nurse is attending to a 7-year-old boy, and his mother informs the nurse that her child is scheduled for CVL insertion tomorrow morning. The child is playing a game on his phone without paying attention to the discussion between his mother and the nurse. The nurse further informs the parent that the child needs to fast from midnight tonight for pre-op preparation. The parent nods to indicate that she understands. The nurse then leaves the room. After the nurse leaves, the mother informs the child that he must fast starting at midnight because the doctor is going to give him an injection in the morning. The child asks why he needs to fast for an injection. The mother says that it is because of the strong medicine that will be injected without explaining the CVL insertion. (Fieldnote, 31)

In contrast to the two previously described scenarios, the third example illustrates how the parent acted as a communication buffer by shielding her child from distressing information, which may have prevented the child from participating in the decision-making process. It can be observed that the child appeared to be dissatisfied with his mother’s explanation and in need of more information, which was evident in his attempts to clarify why he needed to fast. However, the mother did not convey the exact information; she explained that the requirement for fasting was because of the strong medication to be administered by the physician. This situation also shows that minimal efforts were made by the nurse to communicate directly with the child; communication was carried out solely with the parent. The child was expected to comply with the treatment plan without fully understanding why.

## Discussion

According to research, children do not always contribute to care-related communication or decision making. As evidenced by the three scenarios, parents play a crucial role in the communication process as facilitators of communication, communication brokers or communication buffers, with the ability to either facilitate or restrict children’s participation. This finding is consistent with previous studies’ findings, which determined that parental activities are factors that influence children’s active participation (3–10).

It is noteworthy that when the parent facilitates communication between the nurse and child, the child is able to speak and voice preferences directly to the nurse. Nurses often provide direct information to parents, which means that children are often relegated to non-participant status in consultations (6–8). Some parents’ willingness to allow their child to respond to questions directed to them demonstrates the parent’s value and respect for the child. Parents may be aware that they might not know everything about their children’s likes and dislikes, and allow the children to make their own decisions. Parents’ actions in such situations may reflect their appreciation that they do not always know what is best for their children and that children’s preferences may change over time (16). Importantly, a parent’s presence and response as a facilitator of communication allows the child to increase their confidence levels through asking or answering questions, thus being part of the decision-making process. A child’s increased participation is partly due to the parent’s provision of space in the communication (17). This corresponds to the findings of this study (18–20).

Also highlighted in this study is that children use their parents to manage the burden of communication with the nurse. Because of difficulties communicating with nurses, children rely on their parents to be their advocates and/ or interpreters in the communication process (17). Parents dominate the role of ‘translators’ by conveying information to the children in an easy-to-understand manner, which helps children make choices. This finding supports previous studies that describe parents liaising with children in their own language if health care professionals spoke a different language (16, 17). Nevertheless, the findings indicate that parents assuming roles as communication brokers (by translating and repeating information) does not always lead to the promotion of children’s understanding. There were situations in which the parents did not convey all the information to the children. This suggests that it may be challenging for parents to be interpreters in their children’s care. While parent interpreters are of great use to health care professionals, parents cannot be expected to provide complete information to their children on a universal scale.

On other occasions, parents withheld information from their children for some reason. Parents may withhold information selectively, which could influence their children’s participation in the decision-making process. Parents do not always support their children in difficult situations and nurses sometimes inform children about what is going to happen without presenting alternatives or asking for their views. Many children rely on their parents to be advocates and interpreters in the communication process, but parents may act as communication buffers, limiting exposure to potentially worrying information (16, 18). This finding concurs with previous studies that have described parents undertaking the role of communication executive and engaging in information boundary setting (15–17). As a result, children experience difficulties participating in the decision-making process because of a lack of information, a lack of time for discussion and their preferences being ignored. Parents’ actions play a significant role in supporting or hindering their children’s participation (15–17).

## Conclusion

This study provides unique insight into parental roles that can enable or inhibit children’s participation in the decision-making process. The ways in which parents forge communication between nurses and children, and manage communication difficulties by translating and repeating information from the nurse have positive implications for children’s involvement in the decision-making process. On the other hand, if parents filter and shield what children are told, this might restrict children’s engagement in their care and the related decision-making processes, although this may not be problematic for some children. Thus, nurses should safeguard individualised and respectful facilitation from parental involvement that may encourage children’s participation in decision-making processes related to their health care.

## Figures and Tables

**Table 1:** Demographic characteristics of parents (*n* = 21).

Demographic characteristics	Frequency (percentage)
Age (years old)	
30–40	12 (57.1%)
> 40	9 (42.9%)
Gender	
Female	19 (90.5%)
Male	2 (9.5%)
Race	
Malay	16 (76.2%)
Chinese	3 (14.3%)
Indian	2 (9.5%)
Education level	
Primary education	1 (4.8%)
Secondary education	12 (57.1%)
College/University	8 (38.1%)
Diagnosis of child	
Acute lymphocytic leukaemia	15 (71.4%)
Acute myeloid leukaemia	3 (14.3%)
Chronic myeloid leukaemia	3 (14.3%)
Number of children	
≤ 3	13 (61.9%)
> 3	8 (38.1%)
Child’s previous hospitalisation	
Yes	11 (52.4%)
No	10 (47.6%)
